# Urgent surgery for COVID-19–positive pediatric patient

**DOI:** 10.1186/s40981-021-00461-9

**Published:** 2021-07-22

**Authors:** Franchesca Rivera-Calonje, Shiu-Yi Emily Chen, Carl Lo, Sang Le, Makoto Nagoshi

**Affiliations:** Department of Anesthesiology and Critical Care Medicine, Children’s Hospital Los Angeles, University of Southern California, 4650 Sunset Blvd, Los Angeles, CA 90027 USA

**Keywords:** COVID-19, Pediatric anesthesia, Spinal anesthesia, Airway management, Perioperative management

## Abstract

**Background:**

We present a case of COVID-19–positive pediatric patient for urgent urological surgery by spinal anesthesia to avoid aerosolizing procedure.

**Case presentation:**

A 12-year-old, COVID-19–positive boy presented for urgent wound incision and drainage at the circumcision site. Our anesthetic plan consisted of spinal anesthesia with sedation. He was transported from the COVID-19 isolation floor to the negative pressure operating room. He was placed in lateral decubitus position and oxygen was delivered through facemask. Under sedation, spinal anesthesia was achieved at first attempt. The patient maintained spontaneous ventilation without airway intervention. Patient was recovered in the operation room then transported back to the floor.

**Conclusion:**

Spinal anesthesia is a safe alternative to general endotracheal anesthesia for many pediatric urology procedures. Effective team communication and preparation are keys when caring COVID-19–positive patient in perioperative setting to avoid minimize the risk to healthcare providers.

## Introduction

COVID-19 is highly infective and can spread through contact as well as respiratory and airborne transmission. Airborne transmission via aerosolizing procedures can infect individuals more than 6 ft. away [1]. During the Coronavirus Disease 2019 (COVID-19) pandemic, providers have found ways to limit viral transmission without compromising patient care.

Minimizing the risk of transmission is vital to safeguard the well-being of all Health Care Professionals (HCP) battling the COVID-19 global pandemic. The capacity for human-to-human transmission of COVID-19 continues to pose a high-risk to all HCP in the perioperative setting [2]. When caring for a patient with COVID-19, the Anesthesia Patient Safety Foundation (APSF) recommends that prior to exposure to aerosolizing procedures HCP should protect themselves by donning the appropriate personal-protective-equipment (PPE) [3].

Spinal anesthesia achieves both surgical anesthesia and analgesia without airway intervention and can be performed safely and effectively, while minimizing aerosolization [4]. Spinal anesthesia in pediatric patients has, historically, been unpopular among both anesthesiologists and surgeons. Nonetheless, it has been proven effective in lower abdominal, urologic, and lower extremity procedures. Studies have indicated that spinal anesthesia is a suitable alternative to general anesthesia in neonates, young infants, and small adult patients undergoing minor surgery [5].

In a survey conducted by The Society for Pediatric Urology and European Society of Pediatric Urology, it was found that reluctance to accept spinal anesthesia for routine pediatric urologic procedures is multifactorial [6]. The belief that spinal anesthesia is technically challenging, and thereby increases preoperative time, may contribute to surgeon hesitation in incorporating this procedure. To the contrary, Nationwide Children’s Hospital demonstrated that a dedicated team could induce spinal anesthesia in an average of four minutes [7].

The COVID-19 pandemic has led to questions about how to best care for COVID-19 patients perioperatively [8]. Currently, there are no guidelines extant in the literature for the anesthetic management of children with COVID-19.

As such, we present the case of a COVID-19–positive pediatric patient who successfully underwent urgent urological procedure with spinal anesthesia. This offers the first documented report of the use of regional anesthesia as primary anesthetic modality in a COVID-19–positive pediatric patient.

## Case presentation

A 12-year-old Latino male (BMI 25) presented 12 days post-circumcision for urgent revision surgery. The patient had no past medical history, no respiratory symptoms, and tested negative for COVID-19 at the time of his initial surgery. At his follow-up appointment, the pediatric urologist determined he required revision to avoid poor cosmetic outcome. He was subsequently brought to the hospital for incision and drainage of post-operative hematoma.

### Clinical findings

On exam, the child was in clear discomfort and had a large hematoma completely surrounding the penile shaft. In adherence to Children’s Hospital Los Angeles (CHLA) guidelines, the patient underwent nasopharyngeal COVID-19 swab testing prior to proceeding to the operating room (OR). His test returned positive for severe acute respiratory syndrome coronavirus (SARS-CoV). Per institutional policy, though he was asymptomatic, the patient was presumed to be carrying the virus and be high risk for disease transmission. Shortly after his positive result, we learned that the child’s mother also had been infected with COVID-19 and was recuperating at home.

### Timeline

After a risk-benefit discussion with the surgeon, spinal anesthesia with monitored anesthesia care (MAC) was recommended for this urgent procedure with the objective of avoiding instrumentation of the airway and minimizing the risk of airborne viral transmission. A plan was formulated together with nursing to ensure appropriate and effective communication throughout the case.

Once in proper PPE, the anesthesia and nursing teams transported the patient from the COVID-19 isolation floor to the negative pressure OR. He was transported in a wheelchair, donned in a face mask and isolation gown. At the end of the procedure, the recovery room nurse and OR staff transported the patient from the negative pressure OR, back to the COVID-19 isolation floor without complication.

### Diagnostic assessment

The urgency of this case was discussed with the surgical team and it was concluded that postponing surgery because of his COVID–positive status was unacceptable due to his increased risk for long-term sequalae with his complication. A plan was made to mitigate risk to HCP using the following goals: (1) avoid airway instrumentation, (2) minimize airway manipulation, and (3) reduce overall risk of viral airborne transmission. As a successful spinal anesthetic does not require airway instrumentation or general anesthesia, it was especially suited to this urgent procedure [9]. The plan for spinal anesthesia was discussed with the child’s parents in detail. The pediatric urologist lent support in educating the parents on the benefits of spinal anesthesia for this case. The parents later reported the surgeon’s involvement helped make them comfortable with spinal anesthesia for their son.

### Therapeutic intervention

Premedication was not provided until arrival in the OR to avoid potential sedation and respiratory depression that could necessitate airway intervention during transport. Standard anesthesia monitors were placed prior to administration of premedication. Spontaneous ventilation was confirmed with capnography and end-tidal CO^2^ monitoring. Sedation for spinal placement was achieved with propofol infusion at 35 mcg/kg/min and incremental doses of ketamine (24 mg) and dexmedetomidine (16mcg).The patient’s back was prepped and draped in the usual sterile fashion at the level of the L3/L4 interspace. Lidocaine 1% (2 mL) was used to provide local anesthesia to the skin. A Gertie-MarxTM 25-gauge spinal needle was used to access the spinal canal with return of clear cerebrospinal fluid (CSF).

Spinal anesthesia was achieved on the first attempt within 5 min. A total of 2 mL, consisting of bupivacaine 0.75% with dextrose 5% (1.5 mL) and fentanyl PF 25 mcg (0.5 mL) was given. The pediatric urologist then proceeded with their portion of the procedure. Surgical anesthesia was tested prior to incision and no response to noxious stimuli was appreciated. Surgery proceeded for 45 min with the patient breathing spontaneously throughout. There were no anesthetic complications or need for airway manipulation. The child was able to move his feet prior to discharge from the OR.

### Follow-up and outcomes

Adequate preparation and communication proved essential in caring for this patient with COVID-19 [10]. Careful planning, clear communication, and precise anesthetic execution are needed for success. In this case, all parties, including the patient, his parents, the pediatric urologist, and the nursing teams were pleased with the anesthetic care provided for this patient.

### Patient perspective

Post-operation discussion with the parent and patient highlighted the importance of patient-doctor rapport. Both the parent and patient were aware of the perioperative plan which they said served as a form of anxiolysis and facilitated the child’s cooperation. The family and patient were satisfied with the care provided and appreciated the Anesthesiologist’s effort to evaluate and minimize the potential risk of this urgent case.

## Discussion

This is the first documented use of spinal anesthesia as primary anesthetic modality in a pediatric patient with novel COVID-19. Spinal anesthesia achieves both surgical anesthesia and analgesia without airway intervention [11]. This report highlights the importance of effective communication during the COVID-19 pandemic. The anesthetic approach demonstrated in this case effectively balances the safety of both patient and HCP, reducing the risk of spreading COVID-19 while simultaneously providing high-quality patient care. Unnecessary exposure to HCP was minimized by maintaining pediatric surgery left much to be desired. Five articles discussed the clinical findings communication and preparation are key to success when caring for COVID-19–positive patients in the perioperative setting to minimize the risk of viral transmission in a literature search in Spring 2020 [12].

At this time, there is no consensus on anesthetic management of patients with COVID-19. A literature review of 690 articles in PubMed, using the keywords “COVID-19 pediatric surgery” left much to be desired. Five articles discussed the clinical findings associated with COVID-19 infection in children and one article indicated that multisystem inflammatory syndrome, which is common in children with COVID-19, may have cardiac involvement [13, 14].

Advances in the realm of pediatric regional anesthesia continue to evolve with excellent safety profiles described in society guidelines [15]. New data on the safety and efficacy of anesthesia experiences similar phenomenon. Anesthesiologists should be encouraged regional/neuraxial anesthesia in children continues to emerge from all over the world, even in remote settings such as rural Madagascar [16]. As the field of acute pain management and regional anesthesia continues to grow, the field of pediatric regional anesthesia experiences similar phenomenon. Anesthesiologists should be encouraged to advocate and provide education to surgical colleagues as well as patients and their families about the safety and efficacy of regional and neuraxial anesthesia.

Spinal anesthesia is a safe alternative to general endotracheal anesthesia for many pediatric urology procedures [17]. Data from The Ohio State University found that although rare, complications from spinal anesthesia include high spinal level (3.8%), limited analgesic time (2.7%), bradycardia (1.6%), oxygen desaturation (0.6%), and LA toxicity (0.5%). Extremely rare complications include infection and neurologic injury (not some practitioners in performing neuraxial techniques can lead to some hesitancy to permanent). None of these complications were seen in this case [18]. Comfort level of some practitioners in performing neuraxial techniques can lead to some hesitancy to incorporate them into practice. It is important to be aware of the distinctions between pediatric and adult populations when performing spinal anesthesia. Limited analgesia time is common in children and must be taken into consideration [5, 9].

## Conclusion

While the management of pediatric patients with COVID-19 must be individualized, spinal anesthesia should be strongly considered as an alternative to general anesthesia for lower abdominal and lower extremity surgery in COVID-19–positive pediatric patients.

## Data Availability

Not applicable.
